# Subclinical ocular alterations in Graves' disease: The FUMO score, a new tool to predict Graves' orbitopathy progression

**DOI:** 10.1210/clinem/dgag037

**Published:** 2026-02-02

**Authors:** Giulia Lanzolla, Francesca Saba, Silvia Corrias, Filippo Lixi, Giulia Faa, Alessandro Colleo, Chiara Mura, Gian Luigi Canu, Federico Cappellacci, Alberto Cuccu, Giuseppe Giannaccare, Stefano Mariotti, Francesco Boi

**Affiliations:** Department of Medical Sciences and Public Health, Endocrinology Unit, University of Cagliari and University Hospital of Cagliari, Cagliari 09100, Italy; Department of Medical Sciences and Public Health, Endocrinology Unit, University of Cagliari and University Hospital of Cagliari, Cagliari 09100, Italy; Department of Medical Sciences and Public Health, Endocrinology Unit, University of Cagliari and University Hospital of Cagliari, Cagliari 09100, Italy; Eye Clinic, Department of Surgical Sciences, University of Cagliari, Cagliari 09124, Italy; Department of Medical Sciences and Public Health, Endocrinology Unit, University of Cagliari and University Hospital of Cagliari, Cagliari 09100, Italy; Department of Medical Sciences and Public Health, Endocrinology Unit, University of Cagliari and University Hospital of Cagliari, Cagliari 09100, Italy; Department of Medical Sciences and Public Health, Endocrinology Unit, University of Cagliari and University Hospital of Cagliari, Cagliari 09100, Italy; Department of Surgical Sciences, Surgery Unit, University of Cagliari and University Hospital of Cagliari, Cagliari 09100, Italy; Department of Surgical Sciences, Surgery Unit, University of Cagliari and University Hospital of Cagliari, Cagliari 09100, Italy; Eye Clinic, University Hospital of Cagliari, Cagliari 09124, Italy; Eye Clinic, Department of Surgical Sciences, University of Cagliari, Cagliari 09124, Italy; Department of Medical Sciences and Public Health, Endocrinology Unit, University of Cagliari and University Hospital of Cagliari, Cagliari 09100, Italy; Department of Medical Sciences and Public Health, Endocrinology Unit, University of Cagliari and University Hospital of Cagliari, Cagliari 09100, Italy

**Keywords:** Graves' orbitopathy, Graves' disease, thyroid autoimmunity, subclinical ocular disease, thyroid eye disease

## Abstract

**Introduction:**

This observational investigation aimed to assess the accuracy of a novel score in predicting the onset of overt Graves' orbitopathy (GO) in patients with Graves' disease (GD).

**Materials and methods:**

A total of 156 consecutive patients with GD without GO were enrolled. As control group, 45 patients with non-autoimmune hyperthyroidism were included. At baseline, an ophthalmological evaluation was performed, including (1) visual function tests and (2) orbital ultrasound. After 24 months, the occurrence of GO was assessed in all patients.

**Results:**

At baseline, a score from 0 to 3 and a score from 0 to 5 were assigned based on the results of visual function tests and the results of orbital ultrasound, respectively. The scores were combined into an overall FUnctional and MOrphological risk score (FUMO score) (0-8 points), classifying patients as low risk (score 0-2) or medium-high risk (score 3-8). After 24 months, the 2 risk groups were compared for differences in (1) presence/absence of GO and (2) GO activity and severity. Patients in the medium-high risk group developed overt GO more frequently than those in the low-risk group. Additionally, GO was more frequently active and moderate-to-severe in medium-high risk patients. Multiple logistic regression showed that TRAb levels and FUMO were the strongest independent predictors of GO, with higher FT3 and smoking habit levels also conferring increased risk. The model demonstrated good calibration and discrimination (AUC = 0.84; *P* < .0001), with high positive (73%) and negative (72%) predictive value.

**Conclusion:**

Subclinical ocular alterations can predict the progression of GO in patients with GD. The FUMO score, particularly when combined with TRAb and FT3 levels, reliably identifies patients at risk of developing overt GO, supporting its use for early risk stratification.

Graves' orbitopathy (GO) is an autoimmune disorder that affects the orbital fibro-adipose tissue, driven by the interaction between cellular and humoral immunity targeting the thyrotropic hormone (TSH) receptor (TSH-R) and potentially other autoantigens shared by thyroid epithelial cells and orbital fibroblasts (1, 2). The clinical manifestations of GO primarily result from 3 key factors: soft tissue inflammation, excessive glycosaminoglycan production, and adipose tissue expansion (1, 3). Although GO can occur in patients with chronic autoimmune thyroiditis and, in rare cases, in those with no overt thyroid dysfunction—referred to as “euthyroid GO”—it is most commonly associated with Graves' disease (GD) (4). With an estimated incidence of 0.54 to 0.9 cases per 100 000 per year in men and 2.67 to 3.3 cases per 100 000 per year in women (5, 6), GO is considered a relatively rare disease (7). While the majority of patients with GD exhibit no or only mild ocular involvement at diagnosis (7, 8), the overall prevalence of GO across all stages of the disease ranges from 25% to 40% (9). For newly diagnosed GD patients without overt GO, estimating the risk of developing GO could be clinically valuable. Identifying high-risk individuals may lead to a close follow-up of ocular manifestations and help guide hyperthyroidism treatment choices, thereby favoring antithyroid drugs or total thyroidectomy over radioactive iodine, given the potential adverse effects of ^131^I on GO progression (10-12). Additionally, prophylactic selenium supplementation could be considered, as it has been shown to prevent GO worsening in mild cases (13).

A relatively recent study by Wiersinga et al (14) has confirmed 4 independent determinants predictive of GO development in patients with newly diagnosed GD: clinical activity score (CAS), thyroid-binding inhibitory immunoglobulin (TBII) levels, active smoking status, and prolonged duration of hyperthyroid symptoms. The authors prospectively validated the association of these factors with GO incidence within a cohort of hyperthyroid patients devoid of overt GO at study inception. Leveraging these determinants, the PREDIGO predictive score was derived, demonstrating a high negative predictive value (NPV) (0.91). However, the positive predictive value (PPV) was quite low (0.28).

In this context, screening for subclinical ocular alterations in patients with GD without overt GO could offer valuable insights. Indeed, while subclinical ocular changes are recognized in many GD patients without overt GO, their predictive value for disease progression and the development of overt GO remains unclear. The aim of this study was to evaluate the accuracy of a novel score based on subclinical alterations in predicting the progression of GO in patients with GD.

## Methods

### Study design and patient selection

A total of 258 patients with new diagnosis of GD and 124 sex- and age-matched patients with a recent diagnosis of toxic multinodular goiter (TMNG) or toxic adenoma (TA) (control group) were screened. For the GD group, the inclusion criteria were the following: (1) recent diagnosis of GD, namely a history of hyperthyroidism along with previous or present detectable serum thyrotropin receptor binding antibodies (TRAbs); (2) absence of GO defined as no ocular complaints and no clinical signs of GO on examination (no eyelid retraction or edema, no soft-tissue inflammation, no proptosis beyond normal limits, no motility restriction or diplopia, and CAS = 0) (15); (3) written, signed informed consent to data use and sample collection. For the control group, inclusion criteria were the following: (1) recent diagnosis of TMNG or TA and (2) written, signed informed consent. The exclusion criteria for both groups were (1) age < 18 years; (2) GD duration longer than 6 months; (3) presence of corneal dystrophies; (4) local and/or systemic therapy with drugs known to have ocular toxicity; (5) presence of ocular or systemic pathologies that may interfere with assessments (eg, diabetes mellitus, Sjogren's syndrome); (6) previous eye surgery; and (7) recent immunosuppressive therapy for any reason.

Given the absence of pre-existing data on the topic, a formal sample size calculation was not performed before starting the study. However, a post hoc analysis was conducted to confirm the sample size needed for sufficient power.

The study was performed according to the institutional guidelines and with the International Conference on Harmonization Good Clinical Practice guidelines and the principles of the Declaration of Helsinki (1975) and its revised version of 2013. All patients provided informed consent for being included in the study and for the use of their anonymized clinical data for research purposes. Data were anonymized for collection and analysis, avoiding a privacy data breach.

**Figure 1:**
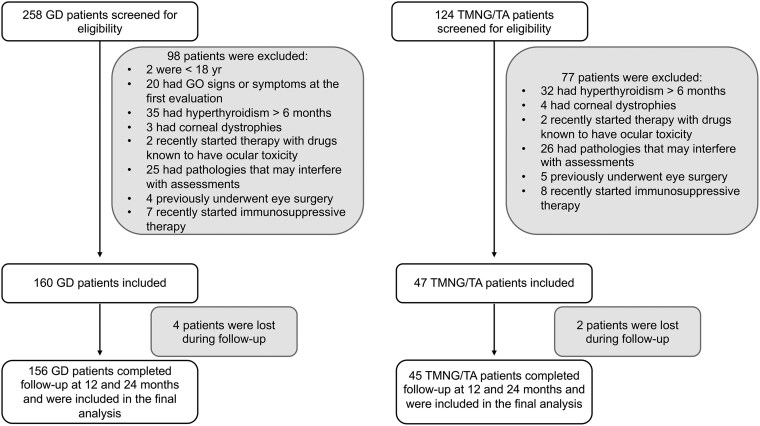
Flow chart of patient selection and follow-up. The diagram illustrates the screening, inclusion, and follow-up of 258 patients with Graves' disease (GD) and 124 patients with toxic multinodular goiter or toxic adenoma (TMNG/TA). After applying predefined exclusion and inclusion criteria, 160 GD and 47 TMNG/TA patients were initially included. During follow-up, 4 GD and 2 TMNG/TA patients were lost, resulting in 156 GD and 45 TMNG/TA patients with complete 24-month follow-up who were included in the final analysis.

A total of 160 GD consecutive patients and 47 sex- and age-matched of TMNG or TA patients who satisfied the inclusion criteria and evaded the exclusion criteria were enrolled. Then, 156 GD patients and 45 TMNG or TA patients who completed the 24 months follow-up period were included in the final analysis (Fig. 1).

Demographic data, including age, gender, smoking habit, GD, previous and current treatment for GD, and general clinical data were collected. The following blood tests were performed in all subjects: free thyroxine (FT4) by chemiluminescence immunoassay (Vitros Immunodiagnostics, Raritan, NJ); free triiodothyronine (FT3) by chemiluminescence immunoassay (Vitros Immunodiagnostics, Raritan, NJ); TSH by immonuochemiluminometric assay (Immulite 2000, Siemens Healthcare, Gwynedd, UK); TRAbs by enzyme-linked immunoassay (ElisaRSR^TM^ TRAb 3rd generation, Cardiff, UK, RRID:AB_2892140). All patients remained euthyroid while receiving methimazole (median dose: 12.5 mg daily) throughout the study.

### Setting

The investigation was carried out at the University of Cagliari (Endocrinology Unit and Ophthalmological Unit) from May 2018 to September 2024. Data were collected and recorded in a database. Database validation procedures included the following: allowed character checks, batch totals, missing records check, cardinality check, digits check, consistency check, control totals, cross-system consistency check, data type check, hash totals, limit check, logic check, presence check, range check, spelling and grammar check, and uniqueness check.

### Outcomes

The primary objective was to develop a novel scoring system based on ocular subclinical alterations identified in GD patients through visual function tests and orbital ultrasound, and to validate it using the control group. The secondary objective was to assess its accuracy in predicting the progression of GO from a clinically silent/subclinical stage to overt GO.

### Sources of data and measurements

At baseline, all patients underwent an endocrinological evaluation along with blood tests and thyroid ultrasound. In addition, an ophthalmological evaluation was performed by the same ophthalmologist, including (1) visual function tests: measurement of intraocular pressure in upgaze, assessment of ocular motility by cover and red glass test; (2) orbital ultrasound.

Intraocular pressure (IOP) was measured using Goldmann applanation tonometry in both the primary gaze position and during upgaze. Measurements were taken to evaluate for restrictive changes associated with GO. In the primary position, IOP was recorded to establish a baseline. During upgaze, IOP was measured to assess for elevation, which can occur due to compression of the inferior rectus muscle, a common site of inflammatory involvement in GO.

The presence of latent ocular motility impairment was assessed using the cover and red glass test. Regarding cover test, patients were instructed to fixate on a distant target while maintaining a primary gaze. Subsequently, each eye was alternately covered with an occluder. Observation of the uncovered eye's movement during the contralateral eye's occlusion allowed for the detection of manifest ocular deviations. Rapid covering and uncovering of each eye facilitated the identification of latent ocular deviations. The test was performed in the primary gaze position and the 8 cardinal positions of gaze to evaluate for ocular deviations in various fields of gaze. The direction and magnitude of ocular movement observed during the test provided information regarding the presence and direction of ocular deviation, potentially indicating the involvement of extraocular muscles. Then, red glass test was performed. Briefly, a red glass was placed in front of the patient's right eye, and the patient was asked to focus on a single white light source directly in front of them, maintaining a primary gaze. The patient was then instructed to look at the light source from various positions of gaze, including the primary position and the 8 cardinal positions. If there was a muscle dysfunction, the patient reported diplopia (perception of 2 lights: white and red). The direction of gaze that resulted in diplopia or showed the greatest separation of the images could suggest which structures were involved.

Orbital US was performed by the same experienced echographer using a B-mode MYLAB 70XV Echo-scan (ESAOTE, Genova, Italy) with a 10 MHz transducer. During the examination, the patients were asked to look straight ahead, maintaining a primary gaze position. The probe was placed on the closed eyelid, on the opposite side of the muscle that was examined. Suitable sections were frozen on the screen when the trans-bulbar muscle stripe appeared as distinctly as possible. The thickest section of all rectus muscles was then measured at the point of greatest enlargement, perpendicular to the muscle axis, using calipers. Although a specific mathematical correction formula or phantom-based calibration has not been applied, all orbital ultrasound examinations were performed with the same machine by a single experienced operator, using a standardized protocol, to minimize measurement error. For each rectus muscle, 3 measurements were obtained on the same image plane, and the mean value was used for analysis, reducing random measurement error. In addition, the equipment underwent regular quality assurance checks according to the manufacturer's recommendations, limiting potential equipment drift over the study period.

Muscles were considered normal if the thickness was <4 mm, thickened if the thickness was between 4 and 6 mm, and very thickened if the thickness was >6 mm.

After 24 months, the occurrence of GO, along with its activity and severity, was assessed in all patients by an ophthalmological evaluation, including (1) Hertel's exophthalmometry (Handaya, Tokyo, Japan); (2) eyelid aperture; (3) CAS; (4) assessment of diplopia evaluated using a Gorman score (16); (5) assessment of the corneal status; (6) presence of lagophthalmos; (7) examination of the fundi; and (8) assessment of visual acuity, measured as best corrected visual acuity (BCVA) in Logarithm of the Minimum Angle of Resolution (LogMar). Symptoms such as photophobia, lacrimation, irritation, blurred vision, burning, itchiness or grittiness and dryness were also investigated.

### Statistical analysis

Continuous data are presented as mean and standard deviation (SD) or median (interquartile range IQR). The Shapiro–Wilk test for normality of distribution was performed on all variables. When appropriate, the following tests were performed: (1) Mann–Whitney U; (2) Fisher exact test. Multivariate analysis was performed by multiple logistic regression. To assess potential multicollinearity among predictors included in the multivariable logistic regression model, we calculated the variance inflation factor (VIF) for each covariate. All variables showed VIF values well below the commonly accepted thresholds for concern (<2), indicating that collinearity was not likely to bias the estimated regression coefficients or their standard errors. A post hoc power analysis for 2 independent proportions (α = .05) was performed using ClinCalc (www.clincalc.com). This analysis showed an achieved power >95%, confirming that the sample size was adequate for both the primary and secondary endpoint. Statistical analysis was conducted by using GraphPad Prism®, version 9.4.1. *P*-values < .05 were considered statistically significant.

## Results

### The development of a novel risk score based on subclinical ocular alterations for predicting Graves’ orbitopathy progression in Graves' disease patients

Baseline demographic and clinical data of the 156 GD patients and 45 GMN/AT patients included in the study are presented in Table 1.

**Table 1:** Demographic and clinical features of patients with Graves' disease (GD)

Features	Graves' disease patients (n = 156)	Multinodular goiter or toxic adenoma patients (n = 45)	*P* value
Gender	Males: 46 (29.7) Females: 110 (70.3)	Males: 13 (28.9) Females: 32 (17.1)	.903
Age (years)	46.01 (11.9)	44.9 (9.7)	.865
Smoking	Never smokers: 75 (48.1) Ex-smokers: 25 (15.8) Current smokers: 56 (36.1)	Never smokers: 20 (44.3) Ex-smokers: 8 (18) Current smokers: 17 (37.7)	.977
Time since diagnosis of hyperthyroidism (months)	4 (3-6)	3.8 (2-6)	.244
TSH (mU/L) NV: 0.4-4	0.67 (0.1-1.3)	0.98 (0.2-1.5)	.454
FT3 (ng/L) NV: 2.7-5.7	4.00 (2.65-6.1)	4.24 (2.98-5.7)	.477
FT4 (ng/dL) NV:0.70-1.70	1.26 (0.93-1.82)	1.36 (0.99-1.62)	.781
TRAbs (UI/L) NV: <1.5	9.83 (3.40-19.97)	0.4 (0.3-0.6)	**<.0001**
Exophthalmometry (mm)	16.5 (1.5)	N/A	N/A
Clinical activity score (points)	0 (0)	N/A	N/A
Eyelid aperture (mm)	9 (1.5)	N/A	N/A
Diplopia	Absent: 156 (100) Intermittent: 0 (0) Inconstant: 0 (0) Constant: 0 (0)	N/A	N/A
Visual acuity (decimals)	1 (0)	N/A	N/A

Data are n (%), mean (SD) or median (IQR). Bold values indicate statistically significant differences (*P* < .05).

Abbreviations: FT3, free triiodothyronine; FT4, free thyroxine; NV, normal values; TRAbs, anti-TSH receptor autoantibodies; TSH, thyrotropic hormone.

To establish a predictive model for the development and severity of GO, a comprehensive scoring system was meticulously developed, incorporating both functional and morphological ophthalmic assessments (Table 2). This system aimed to stratify patients, based on the presence and extent of subclinical ocular alterations, thereby predicting their risk of progressing to clinically significant GO over a 24-month follow-up period.

**Table 2:** Functional and Morphological (FUMO) risk score for Graves' orbitopathy (GO)

Score component	Score range	Examination tool	Interpretation
Visual Function Score	0-3	(1) Intraocular pressure in upgaze (2) Cover test (3) Red glass test	0: No abnormalities → normal visual function. 1: One abnormal test → early subclinical dysfunction. 2: Two abnormal tests → moderate subclinical dysfunction. 3: All 3 abnormal tests → significant subclinical dysfunction.
Orbital ultrasound score	0-5	High-resolution ultrasound assessment of extraocular muscles	0: No thickening or echogenicity changes. 1-4: One point for each thickened muscle (up to 4 muscles). + 1: Additional point if ≥1 muscle >6 mm thick.
FUMO score (FUnctional and MOrphological)	0-8	Sum of visual and ultrasound scores	Combined measure of visual and structural orbital involvement.

The FUMO score combines a visual function score (0-3), derived from a standardized battery of tests (intraocular pressure in upgaze, cover test, red glass test), and an orbital ultrasound score (0-5), based on the number and thickness of extraocular muscles affected on high-resolution orbital ultrasonography. Visual function scores from 0 to 3 reflect increasing degrees of subclinical functional impairment, whereas orbital ultrasound scores from 0 to 5 reflect the extent of extraocular muscle thickening. The combined FUMO score ranges from 0 to 8 and stratifies patients into low-risk (0-2 points; n = 120) and medium–high risk (3-8 points; n = 36) groups.

The scoring system comprised 2 primary components: a visual function score and an orbital ultrasound score. The visual function score, ranging from 0 to 3, was derived from a battery of visual function tests, namely intraocular pressure in upgaze, cover test and red glass test, designed to evaluate the potential latent inflammation and impairment of rectus muscles. A score of 0 indicated the absence of any detectable abnormalities, reflecting optimal visual function. A score of 1 signified the presence of subtle alterations in one visual function tests, suggesting early-stage dysfunction. A score of 2 denoted abnormalities in 2 out of 3 tests, indicating a more pronounced impairment. Finally, a score of 3 represented the presence of abnormalities across all assessed visual function tests, indicating significant, yet subclinical, functional compromise. The specific visual function tests included in this assessment were carefully selected to capture a broad spectrum of potential abnormalities, such as visual acuity, color vision, contrast sensitivity, and visual field analysis. These tests were chosen for their sensitivity in detecting early changes associated with GO, even before the onset of overt clinical manifestations. Each test was standardized and performed under controlled conditions by the same expert ophthalmologist in all patients, to ensure reliability and reproducibility.

The orbital ultrasound score, ranging from 0 to 5, was based on the severity of muscle thickening and the number of extraocular muscles involved, as determined by high-resolution orbital ultrasound. This imaging modality provided detailed cross-sectional views of the orbital structures, allowing for precise measurement of muscle dimensions and identification of subtle changes in muscle echogenicity. A score of 0 indicated no detectable muscle thickening or abnormalities, reflecting normal orbital anatomy. Scores from 1 to 4 represented the presence of muscle thickening, with one point assigned for each muscle involved. An additional point was assigned if the thickening of at least one muscle exceeded 6 mm.

The visual function and orbital ultrasound scores were then combined to generate an overall FUnctional and MOrphological risk score (FUMO score), ranging from 0 to 8 points. Patients were subsequently categorized into 2 risk groups: a low-risk group, comprising patients with scores of 0 to 2 (120 patients), and a medium-high risk group, encompassing patients with scores of 3 to 8 (36 patients). None of the patients in the medium–high risk group had a score above 5, which is somewhat reassuring, suggesting that no cases of GO were misdiagnosed at baseline.

To validate the scoring system, all patients in the control group underwent the same visual function tests and orbital ultrasound assessments. Notably, thyroid function did not differ between GD patients and control group. None of the TMNG or TA patients demonstrated abnormalities in any of the tests. The absence of such alterations in patients with hyperthyroidism of non-GD etiology supports the hypothesis that these subclinical ocular changes are not secondary to hyperthyroidism per se but are intrinsic to GD and potentially linked to an underlying predisposition to develop GO.

### FUMO score: predicting Graves’ orbitopathy severity and activity from subclinical ocular alterations in Graves' disease patients

After a 24-month follow-up period, the 2 risk groups were compared for differences in: (1) presence/absence of GO; (2) GO activity and severity. The comparison was aimed at evaluating the predictive validity of the scoring system. The demographic and clinical data of the 156 GD patients with and without GO at 24 months are presented in Table 3.

**Table 3:** Demographic and clinical features of patients with Graves’ disease (GD) with and without Graves orbitopathy at 24 months

Features	Graves' disease with Graves' orbitopathy (n = 100)	Graves' disease without Graves' orbitopathy (n = 56)	*P*-value
Gender	Males: 29 (29) Females: 71 (71)	Males: 17 (30.36) Females: 39 (69.64)	.890
Age (years)	45.2 (9.8)	46.7 (10.3)	.921
Smoking	Never smokers: 40 (48.1) Ex-smokers: 20 (15.8) Current smokers: 40 (36.1)	Never smokers: 26 (46.4) Ex-smokers: 9 (16.07) Current smokers: 21 (37.5)	.891
Hyperthyroidism duration (months)	28.1 (26.9-30.1)	27.9 (27.6-29.8)	.799
TSH (mU/L) NV: 0.4-4	1.63 (0.9-2.7)	1.72 (1.2-2.6)	.854
FT3 (ng/L) NV: 2.7-5.7	3.81 (2.98-4.1)	4.22 (2.75-4.8)	.678
FT4 (ng/dL) NV:0.70-1.70	1.34 (0.97-1.82)	1.18 (0.93-1.62)	.766
TRAbs (UI/L) NV: <1.5	8.71 (4.40-14.34)	5.4 (3.4-6.6)	**<.0001**
Exophthalmometry (mm)	20 (19-23)	N/A	N/A
Clinical activity score (points)	3 (2-4)	N/A	N/A
Eyelid aperture (mm)	12 (2.5)	N/A	N/A
Diplopia	Absent: 52 (52) Intermittent: 36 (36) Inconstant: 7 (7) Constant: 5 (5)	N/A	N/A
Visual acuity (decimals)	1 (0)	N/A	N/A

Data are n (%), mean (SD) or median (IQR). Bold values indicate statistically significant differences (*P* < .05).

Abbreviations: FT3, free triiodothyronine; FT4, free thyroxine; NV, normal values; TRAbs, anti-TSH receptor autoantibodies; TSH, thyrotropic hormone.

**Figure 2:**
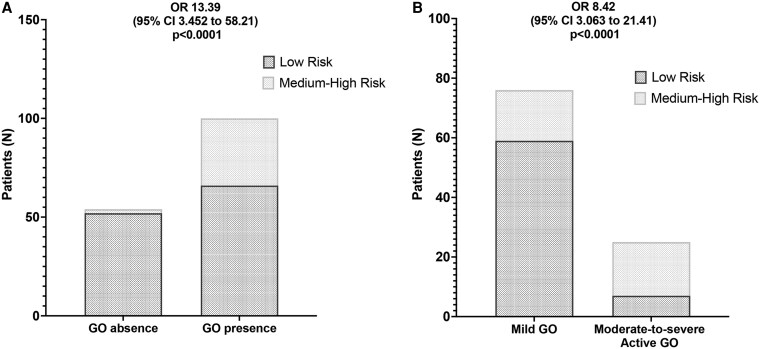
Predictive value of the FUMO score for development, activity and severity of Graves’ orbitopathy (GO) in Graves’ disease (GD) patients. Comparison of (A) GO occurrence (absence vs presence) and (B) occurrence rate of moderate-to-severe active vs mild GO after 24 months of follow-up between low-risk and medium-high-risk GD patient groups, as determined by a composite score of subclinical ocular alterations established at baseline. The composite score, derived from visual function tests and orbital ultrasound, stratified patients into low-risk (score 0-2) and medium-high-risk (score 3-8) groups. Statistical analysis was performed using Fisher's exact test, yielding odds ratios (ORs) with 95% confidence intervals and corresponding *P* values, reported above the graphs.

The results demonstrated a statistically significant association between the medium-high risk group and the development of clinically overt GO. Specifically, patients in the medium-high risk group developed overt GO more frequently than those in the low-risk group (34/36 vs 66/120, OR 13.39, 95% CI 3.45-58.21, *P* < .0001) (Fig. 2A). This finding underscores the predictive power of the FUMO score in identifying patients at increased risk of progressing to overt GO, while it proved to be not very effective in identifying patients who were not at risk of developing GO. Indeed, this new score had a high positive predictive value (PPV: 0.96), while the negative predictive value (NPV) was quite low (0.34).

Furthermore, GO was more frequently moderate-to-severe and active in the medium-high risk group (18/34 vs 7/66, OR 8.42, 95% CI 3.063-21.41, *P* < .0001) (Fig. 2B), indicating that the scoring system not only predicted the development of overt GO but also its activity and severity.

**Figure 3:**
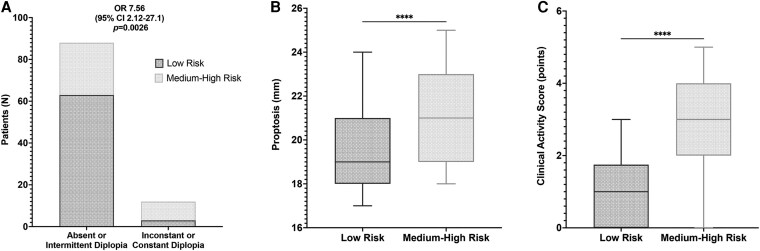
The FUMO score predicts clinical outcomes in Graves’ orbitopathy (GO). Diplopia, proptosis, and Clinical Activity Score (CAS) at 24 months, in Graves' disease patients (GD) stratified by the FUMO risk score. The composite score, derived from visual function tests and orbital ultrasound, stratified patients into low-risk (score 0-2) and medium-high-risk (score 3-8) groups. (A) Number of patients (*y*-axis) with absent or intermittent diplopia vs inconstant or constant diplopia (*x*-axis), comparing the low-risk group (blue bars, FUMO score 0-2) and the medium–high-risk group (red bars, FUMO score 3-8). (B) Proptosis (*y*-axis, mm, Hertel exophthalmometry) in low-risk (blue) and medium–high-risk (red) groups (*x*-axis) as box-and-whisker plots. (C) CAS (*y*-axis, points) in low-risk (blue) and medium–high-risk (red) groups (*x*-axis) as box-and-whisker plots. Statistical analysis was performed using Fisher's exact test (A), yielding both the *P*-value and odds ratio (OR) or Mann–Whitney test (B and C) (*P*-value significance: *P* < .0001 ****).

Consistently, patients in the medium-high risk group also exhibited higher rates of constant or inconstant diplopia (9/34 vs 3/66, OR 7.56, 95% CI 2.12-27.01, *P* < .0026) (Fig. 3A), greater proptosis values (*P* < .0001, Mann–Whitney U = 1396) (Fig. 3B), and higher CAS (*P* < .0001, Mann–Whitney U = 678.5) (Fig. 3C). These findings further validate the clinical relevance of the scoring system in identifying patients with a higher burden of GO-related morbidity.

A detailed analysis of GO progression according to baseline FUMO score is presented in Table 4. The proportion of patients who developed overt GO increased steadily across FUMO categories, from 7.7% in patients with a score of 0 to 93% to 100% in all categories with FUMO ≥3. A similar gradient was observed for disease severity, with the frequency of moderate-to-severe active GO rising from 0% at FUMO 0 to 10.76% at scores 1 to 2 and up to 44.83% to 100% in patients with FUMO scores ≥3 (Table 4).

**Table 4:** Incidence, activity and severity of Graves' orbitopathy (GO) according to FUnctional and MOrphological (FUMO) score categories

FUMO score category	Patients, n	Overt GO occurrence, n (%)	Mild GO n (%)	Moderate-to-severe active GO (%)
0	13	1/13 (7.7)	1/1 (100)	0/1 (0)
1-2	107	65/107 (60)	58/65 (89.24)	7/65 (10.76)
3-4	31	29/31 (93)	16/29 (55.17)	13/29 (44.83)
5-6	3	3/3 (100)	0/3 (0)	3/3 (100)
7-8	2	2/2 (100)	0/2 (0)	2/2 (100)

The number and proportion of Graves' disease (GD) patients who developed overt Graves' orbitopathy (GO) during follow-up, stratified by baseline FUMO score categories (0, 1, 2, 3, 4, ≥5), are presented as follows: for each FUMO category, the table shows the total number patients, as well as the number of patients with any GO, mild GO, and moderate-to-severe active GO allowing visualization of the progressive increase in GO risk and disease burden across higher FUMO scores.

**Figure 4:**
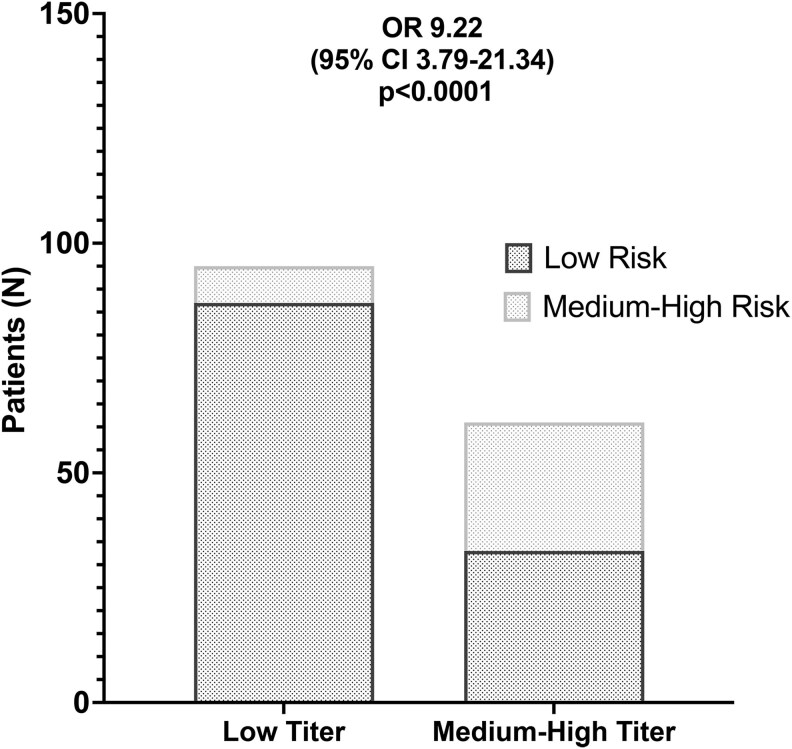
Association of elevated thyroid-stimulating hormone receptor antibody (TRAb) titer with Graves’ orbitopathy (GO) risk stratification. Comparison of TRAb titters (*x*-axis) at the first evaluation between low-risk (blue bars, FUMO score 0-2) and medium-high-risk (red bars, FUMO score 3-8) Graves' disease (GD) patients (*y*-axis), as determined by a composite score of subclinical ocular alterations. TRAb low-titer was defined as 1.75-10 UI/L and TRAb high titer as >10 UI/L. Statistical analysis was performed using Fisher's exact test, yielding both the *P*-value and odds ratio (OR).

Finally, patients in the medium-high risk group were more likely to have elevated (>10 UI/L) TRAb titers at the first evaluation (28/36 vs 33/120, OR 9.22, 95% CI 3.79-21.34, *P* < .0001) (Fig. 4). This association suggests a potential link between immunological activity and the development of GO, further supporting the validity of the scoring system in identifying patients at increased risk.

**Figure 5:**
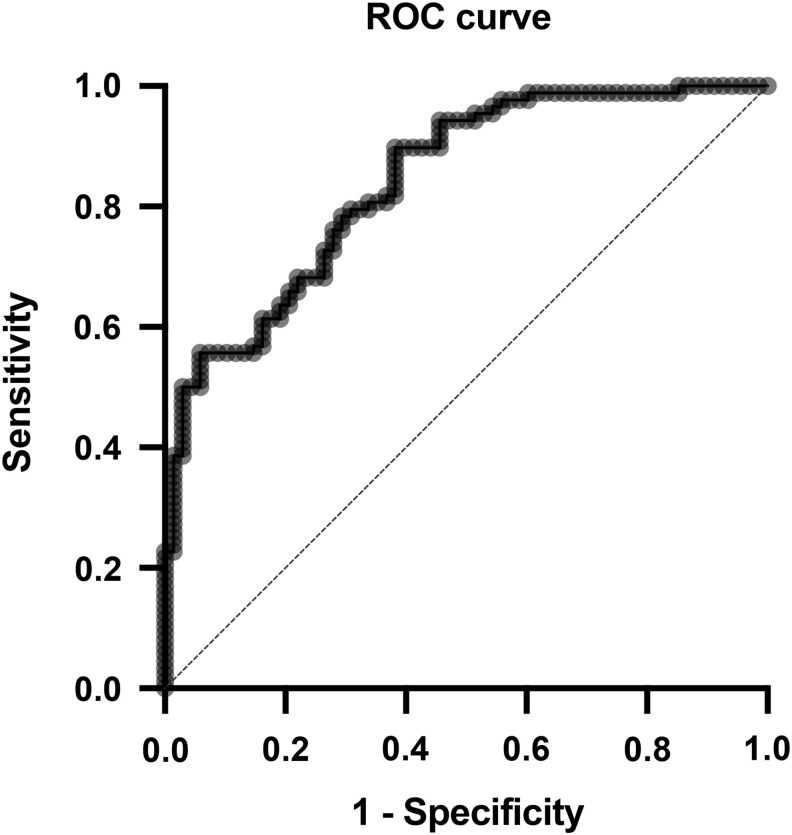
Receiver operating characteristic (ROC) curve for the multivariable logistic regression model predicting the onset of Graves’ orbitopathy (GO) in patients with Graves’ disease (GD). The model included anti-TSH receptor autoantibodies (TRAb) titers, free thyroxine (FT4), free triiodothyronine (FT3), GD duration, and the functional risk score derived from subclinical ocular alterations (FUMO score) as predictors. The area under the ROC curve (AUC) was 0.84 (95% CI 0.78-0.90), indicating good discriminative ability. The model demonstrated sensitivity of 81.8% and specificity of 61.8% at a classification cutoff of 0.5.

To further investigate independent predictors of GO, we performed a multivariable logistic regression analysis including thyroid function parameters (FT3, FT4, TSH), TRAb levels, smoking habit, GD duration, and the FUMO score. The model showed an excellent discriminative ability (AUC 0.84, 95% CI 0.78-0.90) (Fig. 5) and good calibration (Hosmer–Lemeshow *P* = .27). At the cutoff of 0.5, the sensitivity was 81.8% and specificity 61.8%, with a balanced PPV (73%) and NPV (72%). Among the predictors, TRAb levels >10 UI/L (OR 12.9, *P* < .0001) and FUMO score (OR 30.31, *P* < .0001) emerged as the strongest determinants of GO progression, while smoking habit (OR 3.668, *P* = .037) and higher FT3 levels (OR 4.429, *P* = .03) also conferred an increased risk. Conversely, FT4, TSH, and GD duration were not significant (Table 5).

**Table 5:** Predictors of GO: multivariable logistic regression results

Variable	Odds ratio	95% CI profile likelihood	Likelihood ratio	*P*-value
FT3	4.429	1.150-18.66	4.697	**.0302**
FT4	0.124	0.594-1.780	0.008978	.924
Smoking	3.668	0.157-2.81	3.991	**.037**
TSH	0.708	0.449-1.093	2.422	.119
TRAbs	12.99	4.356-50.05	25.78	**<**.**0001**
GD Duration	0.663	0.507-0.850	2.83	.105
FUMO score	30.31	7.620-157.2	28.82	**<.0001**

Bold values indicate statistically significant differences (*P* < .05).

Abbreviations: FT3, free triiodothyronine; FT4, free thyroxine; TRAbs, anti-TSH receptor autoantibodies; TSH, thyrotropic hormone.

## Discussion

The need to identify GD patients at elevated risk of GO progression remains a subject of considerable discussion and ongoing research. Such identification would facilitate the implementation of tailored therapeutic strategies aimed at mitigating disease progression and GO development, as well as enabling more rigorous follow-up to ensure prompt diagnosis and optimized management. Established risk factors for GO progression in GD patients have been recognized for years and include female sex, smoking, radioiodine, duration of hyperthyroidism, and inadequate control of thyroid dysfunction (16). A role of oxidative stress and selenium deficiency has been also reported (17, 18).

Recent investigations have identified novel contributors, such as diabetes mellitus (19) and LDL cholesterol. Indeed, retrospective studies have suggested a protective effect of statin therapy against GO development in GD patients (20, 21). Furthermore, cross-sectional and retrospective analyses have consistently demonstrated a positive correlation between hypercholesterolemia, particularly elevated LDL-C, and both GO occurrence and CAS (22, 23). Interestingly, high LDL-C levels have been associated with diminished response to intravenous glucocorticoid treatment (24). Consequently, the EUGOGO guidelines recommend considering the correction of hypercholesterolemia in GO management (3).

Notably, the PREDIGO study proposed for the first time a predictive score for the occurrence and progression of GO in GD patients (14). Although this study represented a significant advancement in this area, showing a heightened capacity to identify GD patients unlikely to manifest GO, the precise prediction of those who will develop GO remains a clinical challenge.

In this context, the clinical significance of subclinical ocular alterations in GD patients is often underestimated. To address this knowledge gap, we conducted a prospective study to evaluate the occurrence of such alterations in GD patients. Based on the presence and combination of various morphological and functional ocular abnormalities, GD patients were stratified into low-risk and medium-high-risk categories. At the end of a 24-month follow-up period, a statistically significant higher prevalence of GO was observed in the medium-high-risk patient group compared to the low-risk group, with a PPV of 0.96. Notably, the scoring system developed in this study not only proved effective in predicting the overall risk of progression from a subclinical stage to overt GO but also showed a high predictive accuracy for identifying patients at increased risk of developing moderate-to-severe and active forms of the disease. This highlights its potential clinical utility not only in early risk stratification but also in guiding closer monitoring and timely intervention for those most likely to experience more aggressive disease. Furthermore, by detailing GO outcomes across individual FUMO categories, rather than only dichotomizing patients into low- and medium–high-risk groups, we highlight a clear dose–response relationship between the FUMO score and both the incidence and severity and activity of GO. Even modest increases in the FUMO score were associated with a marked rise in GO risk, while higher scores (≥3) identified subgroups in which the risk of developing overt and moderate-to-severe active GO was significantly higher, underscoring the potential value of FUMO as a graded risk-stratification tool in GD.

The underutilization of orbital ultrasound represents a potential limitation for the widespread implementation of the FUMO score. While alternative imaging modalities such as MRI and CT are well established in the assessment of GO, their routine use is generally reserved for selected clinical scenarios, including diagnostic uncertainty, severe disease, or surgical planning. Due to higher costs, limited accessibility, and, in the case of CT, exposure to ionizing radiation, these techniques are not suitable for systematic risk stratification or longitudinal monitoring. Therefore, although orbital ultrasound is currently not widely used in clinical practice and has been superseded by more advanced imaging, it may provide significant insight into the screening of subclinical ocular alterations in GD patients, particularly when combined with functional tests. Importantly, none of the TMNG or TA patients in the control group exhibited abnormalities on either ultrasound or visual function tests, thereby supporting the concept that subclinical ocular alterations are primarily driven by GD-specific autoimmunity, rather than by simple hyperthyroidism, given the similar FT3 and FT4 levels observed in the control group. These findings reinforce the reliability and specificity of the proposed scoring system in detecting subclinical ocular alterations associated with GD and GO. Interestingly, Lixi et al (25) recently reported that patients with inactive GO may present subclinical extraocular muscles dysfunction and morphological changes, which might not be apparent through routine ocular examinations. In their investigation, the authors demonstrated that the red glass test was effective in detecting latent diplopia, highlighting its utility in identifying subtle ocular motility issues and subclinical muscle involvement. Taken together, these results would suggest that comprehensive evaluations combining functional tests like the red glass test and imaging are essential for early detection of GO-related abnormalities, enabling tailored and prompt management and improving patient outcomes.

Indeed, in our study, the integration of morphological and functional assessments of ocular motility in GD patients appears to offer a valuable and non-invasive tool for identifying those at higher risk of GO progression. These patients may be submitted to a close follow-up of ocular manifestations and may benefit from selenium integrative therapy and topic treatment to potentially prevent or at least mitigate GO occurrence. Furthermore, for medium-high risk patients identified using this novel score, treatment with MMI or thyroidectomy, rather than RAI, is likely more appropriate. Additionally, should RAI be necessary, these patients may benefit from corticosteroid prophylaxis, even in the absence of traditional risk factors typically used to determine the need for oral corticosteroid prophylaxis following RAI.

Notably, we observed a significantly elevated prevalence of patients experiencing GO in our cohort. This finding may be contributed, at least in part, to the setting of the study. Indeed, Sardinian population shows a distinct genetic profile and increased autoimmune disease prevalence compared to broader populations (26, 27). Consequently, differential disease progression in Sardinian GD patients cannot be excluded. Since our cohort was derived from a single geographical area, thereby limiting the generalizability of the results, caution is warranted when extrapolating the performance of the FUMO score to populations with different demographic, clinical, or healthcare characteristics. External validation in independent and geographically diverse cohorts is needed before widespread adoption.

To our knowledge, this is the first investigation to propose a scoring system, based on comprehensive ophthalmic evaluation of subclinical alterations. This novel score has been designed to identify ocular subclinical alterations facilitating GD patient categorization into discrete risk strata with a markedly high positive predictive value (PPV). In comparison to the PREDIGO study, which showed a high NPV and low PPV, the FUMO score proposed in this study has high PPV and low NPV. This finding suggests the potential utility of a unified scoring model integrating demographic (smoking), disease-specific (hyperthyroid symptom duration, TRAbs, thyroid function), and ocular parameters. In line with these considerations, our multivariable logistic regression analysis showed that TRAbs, FT3, smoking habit and the FUMO score have particularly strong associations with GO occurrence. The model demonstrated good calibration and robust discriminative ability, with an AUC of 0.84, underscoring its reliability in identifying patients at higher risk. These results complement and extend the predictive capacity of the novel FUMO scoring system by confirming that the integration of serological, disease-specific, and ocular functional parameters provides a more comprehensive risk assessment. Although these findings suggest that the FUMO score might improve the predictive performance of previously proposed tools such as PREDIGO, we did not directly evaluate the PREDIGO score in our cohort, and therefore no formal head-to-head comparison can be drawn. Moreover, the PREDIGO score was developed and validated in a large multicenter cohort, which may reduce center-specific biases that can affect single-center studies like ours.

Overall, our findings can be interpreted within the broader framework of preclinical and subclinical autoimmune diseases. In several autoimmune conditions, immunological activation and tissue remodeling are known to precede the onset of overt clinical manifestations by months or years, giving rise to a phase characterized by subclinical structural or functional alterations. GO appears to follow a similar paradigm. Previous studies (28, 29) have shown that inflammatory activation of orbital fibroblasts and adipogenesis can precede overt clinical signs, leading to subtle changes in extraocular muscles and orbital fat detectable only by imaging. In this context, the identification of such subclinical alterations may reflect an early phase of disease progression, occurring before the development of clinically apparent GO. In this context, the subclinical ocular alterations captured by the FUMO score are likely to reflect early pathogenic changes rather than mere measurement noise and may represent an intermediate stage in the transition from a clinically silent autoimmune process to overt, active and sometimes moderate-to-severe GO. This interpretation reinforces the biological plausibility of imaging- and function-based scores such as FUMO to identify GD patients at increased risk of clinically significant GO. Compared with the PREDIGO score, which emphasized negative predictive value, our findings highlight that combining subclinical ocular alterations with TRAbs and clinical variables enhances the identification of GD patients likely to develop overt GO, offering a tool with both strong clinical relevance and potential utility for guiding early intervention strategies. Larger, multicenter studies are warranted to confirm our results and propose and validate a robust predictive score to be introduced in clinical practice.

## Data Availability

All data generated or analysed during this study are included in this published article.

## References

[1] Bahn RS . Current insights into the pathogenesis of Graves’ ophthalmopathy. Horm Metab Res. 2015;47(10):773‐778. 26361262 10.1055/s-0035-1555762

[2] Lanzolla G, Marino M, Menconi F. Graves disease: latest understanding of pathogenesis and treatment options. Nat Rev Endocrinol. 2024;20(11):647‐660. 39039206 10.1038/s41574-024-01016-5

[3] Bartalena L, Kahaly GJ, Baldeschi L, et al The 2021 European Group on Graves’ orbitopathy (EUGOGO) clinical practice guidelines for the medical management of Graves’ orbitopathy. Eur J Endocrinol. 2021;185(4):G43‐G67. 34297684 10.1530/EJE-21-0479

[4] Viola N, Colleo A, Casula M, Mura C, Boi F, Lanzolla G. Graves’ disease: is it time for targeted therapy? A narrative review. Medicina (Kaunas). 2025;61(3):500. 40142311 10.3390/medicina61030500 PMC11943693

[5] Abraham-Nordling M, Bystrom K, Torring O, et al Incidence of hyperthyroidism in Sweden. Eur J Endocrinol. 2011;165(6):899‐905. 21908653 10.1530/EJE-11-0548

[6] Laurberg P, Berman DC, Bulow Pedersen I, Andersen S, Carle A. Incidence and clinical presentation of moderate to severe Graves’ orbitopathy in a Danish population before and after iodine fortification of salt. J Clin Endocrinol Metab. 2012;97(7):2325‐2332. 22518849 10.1210/jc.2012-1275 PMC3387399

[7] Bartalena L, Piantanida E, Gallo D, Lai A, Tanda ML. Epidemiology, Natural history, risk factors, and prevention of Graves’ orbitopathy. Front Endocrinol (Lausanne). 2020;11:615993. 33329408 10.3389/fendo.2020.615993 PMC7734282

[8] Tanda ML, Piantanida E, Liparulo L, et al Prevalence and natural history of Graves’ orbitopathy in a large series of patients with newly diagnosed graves’ hyperthyroidism seen at a single center. J Clin Endocrinol Metab. 2013;98(4):1443‐1449. 23408569 10.1210/jc.2012-3873

[9] Schuh A, Ayvaz G, Baldeschi L, et al Presentation of Graves’ orbitopathy within European Group On Graves’ Orbitopathy (EUGOGO) centres from 2012 to 2019 (PREGO III). Br J Ophthalmol. 2024;104:294‐300. 10.1136/bjo-2022-322442 PMC10850632 36627174

[10] Bartalena L, Marcocci C, Bogazzi F, et al Relation between therapy for hyperthyroidism and the course of Graves’ ophthalmopathy. N Engl J Med. 1998;338(2):73‐78. 9420337 10.1056/NEJM199801083380201

[11] Tallstedt L, Lundell G, Torring O, et al Occurrence of ophthalmopathy after treatment for Graves’ hyperthyroidism. The Thyroid Study Group. N Engl J Med. 1992;326(26):1733‐1738. 1489388 10.1056/NEJM199206253262603

[12] Acharya SH, Avenell A, Philip S, Burr J, Bevan JS, Abraham P. Radioiodine therapy (RAI) for Graves’ disease (GD) and the effect on ophthalmopathy: a systematic review. Clin Endocrinol (Oxf). 2008;69(6):943‐950. 18429949 10.1111/j.1365-2265.2008.03279.x

[13] Marcocci C, Kahaly GJ, Krassas GE, et al Selenium and the course of mild Graves’ orbitopathy. N Engl J Med. 2011;364(20):1920‐1931. 21591944 10.1056/NEJMoa1012985

[14] Wiersinga W, Zarkovic M, Bartalena L, et al Predictive score for the development or progression of Graves’ orbitopathy in patients with newly diagnosed Graves’ hyperthyroidism. Eur J Endocrinol. 2018;178(6):635‐643. 29650691 10.1530/EJE-18-0039

[15] Bartalena L, Baldeschi L, Boboridis K, et al The 2016 European Thyroid Association/European Group on Graves’ orbitopathy guidelines for the management of Graves’ orbitopathy. Eur Thyroid J. 2016;5(1):9‐26. 27099835 10.1159/000443828 PMC4836120

[16] Bartalena L, Tanda ML. Current concepts regarding Graves’ orbitopathy. J Intern Med. 2022;292(5):692‐716. 35604323 10.1111/joim.13524 PMC9796560

[17] Lanzolla G, Marcocci C, Marino M. Oxidative stress in Graves disease and graves orbitopathy. Eur Thyroid J. 2020;9(Suppl. 1):40‐50. 10.1159/000509615 PMC7802440 33511084

[18] Lanzolla G, Marinò M, Marcocci C. Selenium in the treatment of Graves’ hyperthyroidism and eye disease. Front Endocrinol (Lausanne). 2021;11:608428. 33574798 10.3389/fendo.2020.608428 PMC7870989

[19] Le Moli R, Muscia V, Tumminia A, et al Type 2 diabetic patients with Graves’ disease have more frequent and severe Graves’ orbitopathy. Nutr Metab Cardiovasc Dis. 2015;25(5):452‐457. 25746910 10.1016/j.numecd.2015.01.003

[20] Stein JD, Childers D, Gupta S, et al Risk factors for developing thyroid-associated ophthalmopathy among individuals with Graves disease. JAMA Ophthalmol. 2015;133(3):290‐296. 25502604 10.1001/jamaophthalmol.2014.5103 PMC4495733

[21] Nilsson A, Tsoumani K, Planck T. Statins decrease the risk of orbitopathy in newly diagnosed patients with Graves disease. J Clin Endocrinol Metab. 2021;106(5):1325‐1332. 33560351 10.1210/clinem/dgab070

[22] Sabini E, Mazzi B, Profilo MA, et al High serum cholesterol is a novel risk factor for Graves’ orbitopathy: results of a cross-sectional study. Thyroid. 2018;28(3):386‐394. 29336220 10.1089/thy.2017.0430

[23] Lanzolla G, Sabini E, Profilo MA, et al Relationship between serum cholesterol and Graves’ orbitopathy (GO): a confirmatory study. J Endocrinol Invest. 2018;41(12):1417‐1423. 29923059 10.1007/s40618-018-0915-z

[24] Lanzolla G, Sabini E, Leo M, et al Statins for Graves’ orbitopathy (STAGO): a phase 2, open-label, adaptive, single centre, randomised clinical trial. Lancet Diabetes Endocrinol. 2021;9(11):733‐742. 34592164 10.1016/S2213-8587(21)00238-2

[25] Lixi F, Cuccu A, Giannaccare G, et al Subclinical ocular motility dysfunction and extraocular muscle changes in inactive Graves’ orbitopathy. J Pers Med. 2024;14(8):848. 39202039 10.3390/jpm14080848 PMC11355160

[26] Songini M, Mannu C, Targhetta C, Bruno G. Type 1 diabetes in Sardinia: facts and hypotheses in the context of worldwide epidemiological data. Acta Diabetol. 2017;54(1):9‐17. 27639869 10.1007/s00592-016-0909-2

[27] Olivieri A, Pinna G, Lai A, et al The sardinian autoimmunity study. 4. Thyroid and islet cell autoantibodies in sardinian pregnant women at delivery: a cross-sectional study. J Endocrinol Invest. 2001;24(8):570‐574. 11686538 10.1007/BF03343896

[28] Enzmann DR, Donaldson SS, Kriss JP. Appearance of Graves’ disease on orbital computed tomography. J Comput Assist Tomogr. 1979;3(6):815‐819. 583152

[29] Villadolid MC, Yokoyama N, Izumi M, et al Untreated Graves’ disease patients without clinical ophthalmopathy demonstrate a high frequency of extraocular muscle (EOM) enlargement by magnetic resonance. J Clin Endocrinol Metab. 1995;80(9):2830‐2833. 7673432 10.1210/jcem.80.9.7673432

